# Use of chemotherapy at the end of life in Turkey

**DOI:** 10.1186/1472-684X-13-51

**Published:** 2014-11-19

**Authors:** Sema Sezgin Goksu, Seyda Gunduz, Dilek Unal, Mukremin Uysal, Deniz Arslan, Ali M Tatlı, Hakan Bozcuk, Mustafa Ozdogan, Hasan S Coskun

**Affiliations:** Department of Medical Oncology, Kayseri State Hospital of Research and Education, Kayseri, Turkey; Department of Medical Oncology, Akdeniz University, Faculty of Medicine, Antalya, Turkey; Department of Radiation Oncology, Kayseri State Hospital of Research and Education, Kayseri, Turkey

**Keywords:** Chemotherapy, End of life, Terminally ill, Cancer

## Abstract

**Background:**

An increasing number of patients receive palliative chemotherapy near the end of life. The aim of this study is to evaluate the aggressiveness of chemotherapy in Turkish individuals near the end of life.

**Methods:**

Patients diagnosed with solid tumors and died from 2010 to 2011 in the medical oncology department of Akdeniz University were included in the study. Data about the diagnosis, treatment details and imaging procedures were collected.

**Results:**

Three hundred and seventy-three people with stage IV solid tumors died from 2010 to 2011 in our clinic. Eighty-nine patients (23.9%) patients underwent chemotherapy in the last month of life while 39 patients (10.5%) received chemotherapy in the last 14 days. The probability of undergoing chemotherapy in the last month of life was influenced by: age, ‘newly diagnosed’ patients, and performance status. There was no significant association of chemotherapy in the last month of life with gender and tumor type. Having a PET-CT scan did not alter the chemotherapy decision.

**Conclusion:**

In conclusion, chemotherapy used in the last month of life in a tertiary care center of Turkey is high. Increasing quality of life should be a priority near the end of life and physicians should consider ceasing chemotherapy and direct the patient to early palliative care.

## Background

Chemotherapy plays an important role in the treatment of advanced cancer. Physicians offer patients chemotherapy not only as cure, but for symptom palliation and prolonging survival. Increasing number of patients receive palliative chemotherapy near the end of life [1, 2]. Although there is evidence that the use of chemotherapy near the end of life is not related to its likelihood of providing benefit [3], medical decisions making on ceasing chemotherapy is very difficult since specific guidelines to help physicians with this topic are not available.

Inappropriate use of chemotherapy in the palliative setting may have negative consequences both for the patients and the health care system. Patients are faced with the risk of being administered a therapy, that may be toxic and potentially life-threatening, which may result in a poorer quality of life [4]. Despite the cost-effectiveness issue, ‘the overuse’ of chemotherapy at or near death is also a poor quality measurement of end of life care [5, 6].

High cost imaging modalities (CT: computerized tomography/MRI: magnetic resonance imaging /PET: positron emission tomography) are often used to determine the extent of disease, and may affect decisions about continuing or changing the treatment, or hospice referral at the end of life [7]. However Dinan et al. reported that high cost imaging is not correlated with the de-escalation of aggressive care [8].

Currently there are only a few palliative care units in Turkey [9, 10]. Most of the hospitals, even the university cancer centers, do not have a palliative care unit and patients are treated by the medical oncologists until death. There are no hospices. To our knowledge, there is no data in the medical literature concerning chemotherapy usage at the end of life in the Turkey. So, we conducted a retrospective study to evaluate the aggressiveness of chemotherapy and its associates at the end of life in a tertiary cancer center in Turkey.

## Methods

Patients diagnosed with solid tumors and died from 2010 to 2011 in the medical oncology department of Akdeniz University were included in the study. Akdeniz University is one of the biggest medical centers of the country, and we welcome many patients from the other cities. Like many other University hospitals in Turkey, our institution does not have a palliative care unit and patients are treated by the oncology team until death.

Patients with stage IV solid tumors and who died in the inpatient medical oncology clinics of Akdeniz University were included in the study. Hematologic malignancies that were treated in the hematology service ward were not included. The data collected for this study included demographic data, diagnosis, stage of the disease, whether chemotherapy was given, the time that the last course of chemotherapy was given, performance status at the time of chemotherapy, the time and duration of last hospitalization, imaging studies (CT, MRI, PET-CT), treatments, and the cause of death. Patients were described as ‘newly diagnosed’ if they had cancer diagnosis in the last 2 months of life. All the patients signed an informed consent about using their data with scientific purposes at the time of hospitalization.

Descriptive statistics methods were used for the statistical analysis of the data. The factors associated with chemotherapy at the end of life were analyzed using the multivariate logistic regression model in which we included (in a backward-Wald manner) all the significant variables from the univariate analysis. Mann–Whitney U test was used to compare the time between last chemotherapy and death between newly diagnosed and other patients. A p value of <0.05 was considered significant.

The study is approved by the Ethics Committee of Antalya Research and Training Hospital by reference number 2014–203.

## Results

Three hundred and seventy-three people with solid tumors died from 2010 to 2011 in our clinic. The median age was 60 years (with a range of 20–90). We identified 133 patients (35.7%) ≥65 years old and 240 patients (64.3%) <65 years old. There were 229 men (61.4%), and 144 women (38.6%). The most common diagnoses were non-small cell lung cancer (25.5%), pancreas and biliary tract cancers (11.3%), colon cancer (8.6%), gastric-esophageal cancer (8.6%), gynecologic cancers (7.5%) and breast cancer (6.7%). Seventy four (19.8%) patients were ‘newly diagnosed’. Eight-nine patients (23.9%) underwent chemotherapy in the last month of life and 39 patients (10.5%) were treated in the last 14 days (Table 1).

**Table 1:** **Patient characteristics**

Characteristic n(%)	
Age	
<65 years old	240 (64.3%)
≥65 years old	133 (35.7%)
Sex	
Men	229 (61.4%)
Women	144 (38.6%)
Tumor type	
NSCLC	95 (25.5)
Pancreas and biliary tract	42 (11.3%)
Gastric-esophagus	32 (8.6%)
Colon-rectum	32 (8.6%)
Gynecologic	28 (7.5%)
Breast	25 (6.7%)
Bladder	15 (4%)
Prostate	15 (4%)
Head and neck	14 (3.8%)
SCLC	14 (3.8%)
Primary X	13 (3.5%)
Sarcoma	11 (2.9%)
RCC	10 (2.7%)
GBM	10 (2.7%)
Testis	5 (1.3%)
Melanoma	4 (1.1%)
HCC	4 (1.1%)
M. Mesothelioma	3 (0.8%)
Thymic carcinoma	1 (0.3%)
ECOG	
2	18 (4.8%)
3	154 (41.3%)
4	201 ( 53.9%)
Newly diagnosed	
Yes	74 (19.8%)
No	229 (80.2%)
Chemotherapy in the last month of life	
Yes	89 (23.9%)
No	284 (76.1%)
Chemotherapy in the 14 days of life	
Yes	39 (10.5%)
No	334 (89.5%)
Imaging studies in the last month of life	
High cost imaging in the last month of life (CT/MRI/PET-CT)	
Yes	186 (49.9%)
No	187 (50.1%)
PET- CT in the last month of life	
Yes	42 (11.3%)
No	331 (88.7%)
Cause of death	
Disease Progression	303 (81%)
Renal/hepatic failure*	18 (4.8%)
Sepsis/febril neutropenia	33 (8.8%)
Bleeding	12 (3.2%)
Others**	7 (1.9%)

NSCLC: non-small cell lung cancer, SCLC: small cell lung cancer, RCC: renal cell carcinoma, GBM: glioblastoma multiforme, HCC: hepatocellular carcinoma, PET-CT: positron emition tomography, ECOG: European Collaborative Oncology Group performance status *organ failure due to primary cancer or metastasis were included in the disease progression. **Coronary heart disease, embolism, cerebrovascular disease.

Univariate and multivariate analyses were performed to identify the factor(s) associated with chemotherapy at the end of life. Variables were classified as the most effective forms in the regression model. Three factors (age group, ECOG performance status, and newly diagnosis status) differed significantly between these groups (p <0.05) in univariate analysis. All of these significant variables in the univariate analysis were included in the multivariate logistic regression to analyze mortality risk (Table 2). The multivariate logistic regression analysis identified that chemotherapy at the end of life was significantly associated with age group, newly diagnose, and ECOG performance status. Probability of undergoing chemotherapy in the last month of life was influenced by: age (patients younger than 65 years were undergoing chemotherapy more often than those who were 65 years old or older), ‘newly diagnosed’ patients (patients diagnosed with cancer in the last two months of life were more likely to have chemotherapy), and ECOG performance status (patients with ECOG performance status ≤2 were undergoing chemotherapy more often than those with ECOG performance status 3–4). There was no significant association of chemotherapy in the last month of life with gender and tumor type (Table 2).

**Table 2:** **Factors associated with the use of chemotherapy in the last month of life**

	Univariate analysis		Multivariate analysis	
Risk factor	OR (95% CI)	***p***value	OR (95% CI)	***p***value
Age group (<65 years or ≥65 years)	1.95 (1.14-3.34)	0.015	1.91 (1.09-3.35)	0.024
Gender (male or female)	1.25 (0.76-2.06)	0.377	-	-
ECOG performance score (2 or 3 or 4)	-	0.009	-	0.034
(2 or 3)	4.73 (1.76-12.74)	0.002	3.78 (1.37-10.38)	0.010
(3 or 4)	1.22 (0.74-2.01)	0.442	1.08 (0.64-1.81)	0.770
Type of cancer	-	0.784	-	-
Newly diagnosed (Yes or No)	1.71 (0.98-3.00)	0.059	1.83 (1.02-3.27)	0.042
PET- CT in the last month (Yes or No)	1.31 (0.64-2.68)	0.461	-	-

OR: odds ratio, CI: confidence interval, ECOG: the Eastern Cooperative Oncology Group.

Half of the patients had an imaging procedure with CT, MRI, or PET- CT in the last month of life. Forty-two patients (11.3%) had PET-CT imaging. The indication for PET-CT was staging in 21 patients, and evaluation of treatment response in 21 patients. However, having a PET-CT scan did not affect the decision of chemotherapy in the last month of life (p = 0.461).

Median time between the last chemotherapy and death was 15 days for the patients who had chemotherapy in the last month of their life. Time between last chemotherapy and death was significantly shorter in newly diagnosed patients (median 12.4 days, range 1–58) compared to other patients (median 25 days, range 0–168) (p = 0.006).

## Discussion

This study showed that 23.9% of patients received chemotherapy during the last month of life, while 10.5% of patients were treated during the last 14 days. Median duration between the last course of chemotherapy and death was 15 days (range 1–30) in the subgroup of the patients who had chemotherapy in the last month of life.

There are various reports in the literature from different parts of the world concerning chemotherapy usage near the end of life. Although the heterogeneity of study populations and design makes direct comparison difficult, the percentage of our patients who had chemotherapy in the last month of life (23.9%) was higher than the reported numbers of 9 to 23% [3, 11–17]. However, there were studies that had reported higher rates of chemotherapy in the last month of life varying between 31–55.6% [18–21]. In our study, 10.5% of patients had chemotherapy in the last 14 days of life. Different rates of chemotherapy in the last 14 days were reported in the literature varying between 3.7- 33.8% [1, 11–13, 20–25]. Earle CC reported in his review that the proportion of patients still receiving chemotherapy within 14 days of death is on the rise. Among the 215,484 patients, 17.0% were still being treated within 2 weeks of death [26]. See Table 3 for rates of chemotherapy usage at the end of life from other countries.

**Table 3:** **Chemotherapy at the end of life: review of the literature**

Author –publication year	Country	Chemotherapy in the last month	Chemotherapy in the last 14 days
Hu W-2013 [22]	Canada		3.7%
Emanuel EJ -2003 [3]	USA	9%	
Zdenskowski N -2013 [15]	Australia	12%	
Keam B-2008 [23]	Korea		5.7%
Gonçalves JF-2008 [11]	Portugal	13%	10%
Adreis F-2011 [13]	Italy	16%	6%
Tang ST -2009 [17]	Taiwan	16%	
Kao S-2009 [12]	England	18%	8%
Liu TW-2012 [16]	Taiwan	17-21%	
Näppä -2011 [14]	Sweden	23%	
Göksu SS* 2014	***Turkey***	***23.9%***	***10.5%***
Frigeri M-2013 [27]	Switzerland	24%	
Yun YH-2007 [18]	Korea	31%	
Earle CC -2004 [1]	USA		15.7%
Barbera L-2006 [24]	Canada		16%
Nakano K -2012 [25]	Japan		20%
Randen M -2013 [19]	Sweden	32%	
Braga S-2007 [20]	Portugal	37%	21%
Sanz Ortiz J-2012 [21]	Spain	55.6%	33.8%

*Results of this study.

Patients younger than 65 years were undergoing chemotherapy more often than those who were 65 years old or older. Age was defined as a predictor of palliative chemotherapy near the end of life in other studies [11, 12, 22–24]. However, age alone is not considered a contraindication for the usage of chemotherapy. Older patients may benefit from chemotherapy similar to younger ones, although they have an increased risk of toxicity [28–31]. In order to plan medical treatment in elderly patients, it is mandatory to practice a comprehensive geriatric assessment that includes evaluation of co-morbidities, functional dependence, socio-economic, emotional and cognitive conditions, an estimate of life expectancy, and recognition of frailty [30].

Newly diagnosed patients were more likely to have chemotherapy in the last month of life. One explanation for the use of chemotherapy within 1 month before death is to treat the previously untreated patients with chemo-sensitive tumors [32]. Of the 23 patients who were newly diagnosed and had chemotherapy in the last month, only 2 of the patients had small cell lung carcinoma, so this cannot be a possible explanation for our study group. Näppä et al. reported that 47.0% of patients who received chemotherapy within the last month were given first line palliative chemotherapy, 20% of these even at the first course of first line [14]. They explained these results with the unpredictable fast progression of disease, increased sensitivity to the side effects, and physician errors in formulating the accurate prognosis. We think one possible explanation for the use of chemotherapy within 1 month of death in newly diagnosed patients can be the feeling ‘to give a chance’ to chemotherapy; it may be easier to cease treatment in the heavily treated ones.

It has been reported that tumor type can be a predictor of chemotherapy at the end of life. Patients with breast cancer [11, 20, 32], lung cancer [11, 32], hematologic malignancies [32], and ovarian and pancreatic tumors [20] were more likely to have chemotherapy near the end of life. There was no association between the administration of chemotherapy in the last month of life and tumor type in our study.

PET-CT is a popular imaging procedure in evaluating cancer. Forty-two patients (11.3%) had PET-CT imaging in the last month of life. Hu et al. had reported that 1.1% of the patients with stage IV breast, lung, colorectal and prostate cancer patients had PET-CT imaging in the last month of life [33]. Interestingly, having a PET-CT scan examination did not alter the chemotherapy decision. It may be reasonable to evaluate the treatment response with PET-CT to decide to continue, change or stop the treatment. But in patients who are not medically fit for chemotherapy staging could be done with other imaging modalities such as CT or MRI (In our country, the cost of PET-CT scan is 10 times that of a CT of thorax-abdomen). Dinan et al. reported that high cost imaging is not correlated with the de-escalation of aggressive care. Hospitals within the top quartile of imaging use generally had decreased odds of hospice utilization and higher likelihood of late hospice enrollment across most cancer types [8]. To our knowledge, there is no data on the cost-effectiveness of PET-CT in the terminally ill cancer patients. So physicians should think again when ordering such an expensive imaging procedure at the end of life.

Usage of chemotherapy at the end of life is also associated with the health care system. It has been reported that hospitals with a palliative care unit were significantly associated with less frequent use of chemotherapy near the end of life [15]. Patients who were not given information about palliative care and were treated by a medical oncologist continue to have chemotherapy near death [8, 34]. The supportive and palliative care unit integration decreases the chemotherapy use in the last 30 days of life [35]. Currently there are only a few palliative care units in Turkey [9, 10]. Most of the hospitals, even the university cancer centers, do not have a palliative care unit and patients are treated by the medical oncologists until death. As our center does not have a palliative care unit, our results should be interpreted from this view. The Turkish Ministry of Health and Cancer Control department implemented ‘The Pallia-Turk Project in 2010’ to improve the palliative care system in Turkey [9], but further work is needed to improve this system.

The present study has some limitations. First, our study was conducted in a university teaching hospital. Earle CC had previously reported that large hospital size is a strong predictor of aggressive care [26]. This situation can cause a selection bias. Second, we don’t have the data of the patients who obtained palliative chemotherapy in the last month and died at home or in another hospital. By adding these patient percentages, chemotherapy use in the last month of life may be higher.

## Conclusion

This is the first study that examined the use of chemotherapy at the end of life in Turkey. Chemotherapy use in the last month of life in a tertiary care center of Turkey seems to be high. Patients who were younger than 65 years old, newly diagnosed, and had good performance status were more likely to receive chemotherapy at the end of life. An aggressive approach to continue chemotherapy at the end of life has no survival benefit [36]. Instead, it may contribute to negative outcomes. Increasing the quality of life should be the first goal of treatment near the end of life and physicians should consider ceasing chemotherapy and direct the patient to palliative care center earlier.
